# A label-free fluorescent peptide probe for sensitive and selective determination of copper and sulfide ions in aqueous systems†

**DOI:** 10.1039/d0ra08788b

**Published:** 2021-02-17

**Authors:** Yadan Zhang, Yunhui Cai, Yonghui He, Qinlu Lin, Jiali Ren, Dongsheng Cao, Lin Zhang

**Affiliations:** National Engineering Laboratory for Rice and Byproduct Deep Processing, Hunan Key Laboratory of Processed Food for Special Medical Purpose, Hunan Key Laboratory of Forestry Edible Resources Safety and Processing, School of Food Science and Engineering, Central South University of Forestry and Technology Changsha 410004 P. R. China zhanglin840514@126.com; Key Laboratory of Chemistry in Ethnic Medicinal Resources, State Ethnic Affairs Commission & Ministry of Education, Yunnan Minzu University Kunming Yunnan 650500 P. R. China; Xiangya School of Pharmaceutical Science, Central South University Changsha 410083 P. R. China

## Abstract

A label free fluorescent peptide probe (HDSGWEVHH) was used for Cu^2+^ and S^2−^ determination in aqueous solution. Our results demonstrated that HDSGWEVHH is highly selective and sensitive for monitoring free Cu^2+^ concentration *via* quenching of the probe fluorescence upon Cu^2+^ binding. The mechanism of the complexation is investigated with Cyclic Voltammetry (CV), ^1^H nuclear magnetic resonance (NMR), electron paramagnetic resonance (EPR) spectroscopy and computational techniques. Theoretical calculation results indicated the binding ratio of the probe to Cu^2+^ is 2 : 1 and the binding constant was obtained as 1.72 × 10 ^8^ M^−1^. Cu^2+^ concentration can be detected with the detection limit of 16 nM. Free Cu^2+^ concentration released from the metallothionein–Cu complex at different pH values was detected. Cu^2+^ concentration in real water and tea samples was also detected, and the results were consistent with the ones monitored by atomic absorption spectrometer. Because of the exceedingly small *K*_sp_ value of CuS (1.27 × 10^−36^), S^2−^ can sequester Cu^2+^ from HDSGWEVHH to restore the tryptophan (W) fluorescence. Thus the HDSGWEVHH–Cu^2+^ complex can also be used for S^2−^ detection. The S^2−^ concentrations can be monitored with a detection limit of 19 nM. The assay is also amenable to measurement of S^2−^ concentration in pure water samples. Thus the probe designed herein is sensitive, label free, low cost, and environmentally friendly for Cu^2+^ and S^2−^ determination in aqueous solutions.

## Abstract 2

A label-free fluorescence “on–off–on” peptide probe for selective determination of Cu^2+^ and S^2−^ in a pure water system.

## Graphical abstract

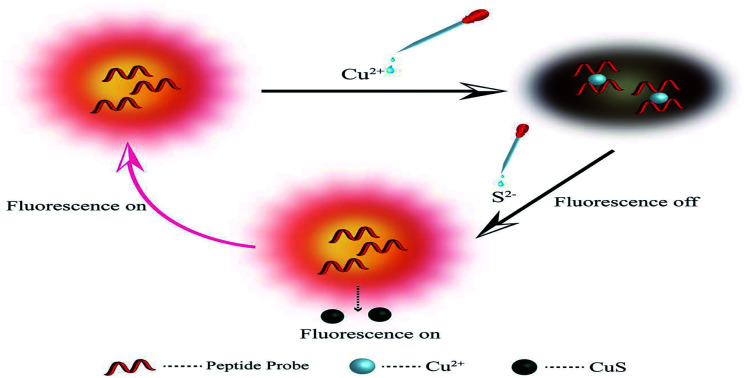

## Introduction

Copper (Cu) is an essential metal for the human body at trace level but its accumulation has been linked to several copper-transport diseases (*e.g.*, Wilson's disease and several neurological disorders).^1^ In cellular milieu, copper is tightly regulated by a variety of proteins (*e.g.* metallothioneins), as free Cu^2+^ can lead to production of reactive oxygen species (through the Fenton-like reaction) and oxidative stress, which could induce damage of cells and human health.^2^ The excess free copper uptake could be from drinking water and foods. Thus the maximum concentrations of copper in drinking water are limited to 1.3 mg kg^−1^ (∼20 μM) and 1.0 mg kg^−1^ (∼15 μM) by the U.S. Environmental Protection Agency (EPA) and China, respectively.^3^ The maximum concentrations in tea are limited to 150 mg kg^−1^ and 60 mg kg^−1^ in the U.S.A. and China, respectively.^4^ Thus monitoring free Cu^2+^ in water or Cu^2+^ released from metallothionein (MT) are important for food safety and biological studies.

Recent studies have suggested that H_2_S is an endogenously produced gaseous signaling compound (gasotransmitter) with importance comparable to that of the other two known endogenous gasotransmitters, nitric oxide (NO) and carbon monoxide (CO).^5,6^ H_2_S contributes to a diverse array of physiological processes, including vasodilation, angiogenesis, oxygen sensing, apoptosis, inflammation, and neuromodulation.^7–9^ But, excessive sulfide (S^2−^) are related with diseases ranging from Alzheimer's disease and Down's syndrome to diabetes and liver cirrhosis.^10–13^ Moreover, S^2−^ as a member of the active sulfur species is an environmental pollutant, which is emitted from the leather industry, pulp removal and refinery.^14^ The excessive S^2−^ could influence the food safety and human health, thus the maximum concentration of S^2−^ in drinking water is limited to 0.48 mg kg^−1^ (∼15 uM) (World Health Organization, WHO).^15^

Recently, a number of different analytical techniques (*e.g.* atomic absorption spectroscopy, inductively coupled plasma atomic emission spectrometry, and electrochemical technique) have been used for determination of copper and sulfide.^16–22^ Nevertheless, the methods generally require time-consuming procedures and the use of expensive, sophisticated instruments. In contrast, some spectroscopic methods were developed.^23–25^ Among them, fluorescence spectroscopy is attractive owing to its simplicity, and high sensitivity. Wu and coworkers synthesized 4-dimethylamino-benzoic acid (2-imidazole formaldehyde)-hydrazide for Cu^2+^ and S^2−^ determination.^15^ The detection limits for Cu^2+^ and S^2−^ determination are 15 nM and 0.12 μM, respectively. Nagano and coworkers reported a Cu^2+^ and S^2−^ determination probe (HSip-1), which consists of a cyclen macrocycle attached to fluorescein.^26^ The detection limits of the HSip-1 method for Cu^2+^ and S^2−^ are both around 10^−6^ M. However, as other organic fluorescent probes, most of the Cu^2+^ coordination compounds need complex synthesis process, and some of them have poor solubility in aqueous solutions and are toxic to cells and human. Thus peptide fluorescence probe were designed for Cu^2+^ and S^2−^ determination. Tang and coworkers developed the dansyl groups labeled tetra-peptide fluorescence probe for detecting Cu^2+^ and S^2−^ in aqueous solutions. The detection limits for Cu^2+^ and S^2−^ are 88 nM and 75 nM, respectively.^27^ A dansyl group labeled peptide fluorescent chemosensor was synthesized for Cu^2+^, Zn^2+^ and S^2−^ determination by the same group. And the detection limits for Cu^2+^ and Zn^2+^ are 78 nM and 82 nM, respectively.^28^ Among these peptide based fluorescent probes for Cu^2+^ and S^2−^ determination, most of them were fluorophore labelled.^29–32^ In our previously work, a label free tetra-peptide was designed for Cu^2+^ detection. The detection limit for Cu^2+^ is 8 nM.^33^ But the binding model and S^2−^ determination characters have not been studied.

To develop a water soluble and label free fluorescent probe for both Cu^2+^ and S^2−^ determination, a mutant amyloid beta (Aβ) peptide (HDSGWEVHH) which is innocuous to cells have been used in the present work as a peptide based fluorescent probe for both Cu^2+^ and S^2−^ detection in aqueous solutions. Aβ peptides are known to interact strongly with Cu^2+^ with a quite large binding constant (*K*_a_ ∼10^11^ M^−1^),^34^ which could result in the high selective binding to Cu^2+^. The fluorescence of the peptide (HDSGWEVHH) could be quenched by Cu^2+^ binding and be recovered by S^2−^. According the fluorescence changes, free Cu^2+^ concentration released from the MT–Cu complex at different pH and Cu^2+^ concentrations in drinking water and tea samples were detected. S^2−^ concentrations in real water samples have also been investigated.

## Experimental

### Chemicals and reagents

HDSGWEVHH was designed and synthesized in our lab with an automatic peptide synthesizer (Symphony Quartet, Tucson, AZ, USA). MT–Zn_7_, isolated form rabbit liver, was purchased from Hunan Lugu Biotechnology Co. (Changsha, China). Sodium sulfide, sodium hydroxide, sulfuric acid, copper sulfate, 5,5′-dithio-bis-(2-nitrobenzoate) (DTNB), 4-(2-hydroxyethyl)-1-piperazinee-thanesulfonic acid (HEPES), and other chemicals were acquired from Sigma Chemicals (St. Louis, MO, USA). Bama tea was purchased from supermarket (Changsha, China). All of the aqueous solutions were prepared using water purified by a Simplicity Water Purification System (Millipore, Billerica, MA, USA) to a resistivity of 18 MΩ cm.

### Peptide synthesis

**Fig. 1:**
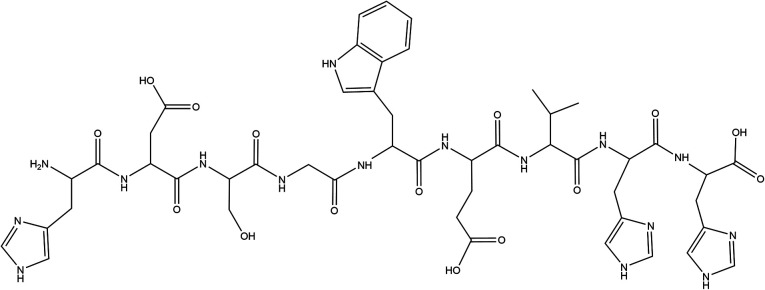
Structure of HDSGWEVHH.

HDSGWEVHH was synthesized *via* solid-phase Fmoc chemistry on a Symphony Quartet peptide synthesizer (Protein Technologies, Tucson, AZ, USA). The Fmoc groups were deprotected with 20% piperidine in dimethylformamide (v/v) after the coupling reaction had proceeded for 30 min. Upon dehydration on a freeze dryer (VirTis Benchtop K, Warminster, PA, USA), the crude product was purified by semipreparative reversed phase (RP) HPLC (Shimadzu 6AD, Columbia, MO, USA) using a column (Jupiter-10-C18-300, 10 mm i.d. × 250 mm) from Phenomenex (Torrance, CA, USA). The eluents were 0.1% trifluoroacetic acid in water (v/v, mobile phase A) and 0.1% trifluoroacetic acid in acetonitrile (v/v, mobile phase B). At a flow rate of 4.75 mL min^−1^, a purification of HDSGWEVHH was accomplished with a linear gradient of 10–65% phase B for 13 min. The purity of the synthesized peptides was verified by HPLC and electrospray-mass spectrometry (ESI-MS) (Fig. S1 and S2†) (Fig. 1).

### Sample preparation

HDSGWEVHH stock solutions (1–2 mM) were prepared by directly dissolving the lyophilized solid samples in 10 mM NaOH. They were then diluted with 10 mM HEPES buffer (pH 7.4) to desired concentration. All concentrations of the peptide solutions were based on UV-vis measurements.

The Cu^2+^ stock solution was prepared by dissolving 1 mM CuSO_4_ in 1 mM H_2_SO_4_ solution. For cation selectivity studies, 1 mM stock solutions of various metal ions were prepared by dissolving FeCl_3_, CaCl_2_, ZnSO_4_, MgCl_2_, MnCl_2_, CoCl_2_, FeCl_2_, Cs_2_SO_4_, NaCl and KCl in 10 mM H_2_SO_4_. Stocks of CrCl_3_, AlCl_3_, CdCl_2_, BaCl_2_ and NiCl_2_ solution (50 μM) were freshly prepared with 50 μM HCl solutions. Stocks of Pb(NO_3_)_2_ and Hg(NO_3_)_2_ solution (50 μM) were freshly prepared with 50 μM HNO_3_ solutions. For anion selectivity studies, 0.1 mM stock solutions of various anions were freshly prepared by dissolving Na_2_S_2_O_3_, Na_2_SO_4_, Na_2_SO_3_, NaHSO_3_, Na_3_PO_4_, NaHCO_3_, NaNO_2_, NaOA_C_, NaF, NaCl, NaBr, and NaI in water. Stock solution of Na_2_S was freshly prepared with deionized (DI) water (Na_2_S was used as a sulfide source in all experiments). All the stock solutions were diluted with 10 mM HEPES buffer (pH 7.4) to desired concentration.

To prepare the MT–Cu solution, the commercial lyophilized powder of MT–Zn_7_ was first dissolved in HCl (pH 2) to a final concentration of 1 mg mL^−1^. The resulting solution was incubated at 4 °C for 12 h, and then centrifuged with the 3000 Da cutoff (YM-3) Millipore membrane (Millipore Billerica, MA, USA) at 13 000 rpm while incubating at 4 °C for 30 min. The supernatant was used as the apo–MT solution. Complete removal of Zn^2+^ from MT–Zn_7_ was confirmed by the disappearance of the characteristic absorption of MT–Zn_7_ at 250 nm.^35^ When the apo–MT was obtained, CuSO_4_ (1 mM) was added into the apo–MT containing solution. The final concentrations of MT and Cu^2+^ both were 100 μM, pH is around 6. Owing to the equal amount of MT and Cu^2+^, the MT–Cu was formed as the final complex. All the aqueous solutions for MT–Cu preparation were prepared using DI water, and purged with nitrogen gas to prevent oxidation of free thiol groups on apo–MT.

### UV-visible spectroscopy

UV-vis measurements were performed using a UV-2450 spectrometer (Shimadzu, Japan). The concentration of soluble peptides were determined according to absorbance at 280 nm and using extinction coefficients of tryptophan (*ε*_279_ = 5400 cm M^−1^) and tyrosine (*ε*_276_ = 1410 cm M^−1^) for HDSGWEVHH and Aβ(1–16), respectively.^36^ The apo–MT concentration was determined by assaying thiol groups with Ellman's reagent, DTNB.^37^

### Fluorescence spectroscopy

Fluorescence measurements of HDSGWEVHH peptide solutions were carried out at room temperature with a Hitachi F-4600 spectrofluorometer (Hitachi, Japan). An excitation wavelength (*λ*_ex_) of 280 nm was used, and the emission intensity were recorded at 304 nm and 356 nm for Aβ(1–16) and HDSGWEVHH, respectively. The entrance and exit slits both were 10 nm. Fluorescence measurements were performed three times, and the standard deviation was plotted as error bar.

The quantum yield (*ϕ*) of Aβ(1–16) and HDSGWEVHH were determined by the following expression:^33^1
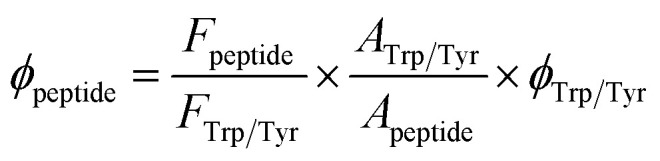
where *F*_peptide_ and *F*_Trp/Try_ are the fluorescence emission intensities of the HDSGWEVHH or Aβ(1–16) peptides and the amino acid reference, which is tryptophan (Trp) or tyrosine (Tyr) dissolved in 10 mM HPEPS buffer (pH 7.4). *A*_peptide_ and *A*_Trp/Tyr_ are the absorbance values at 280 nm of the HDSGWEVHH or Aβ(1–16) peptide samples and the tryptophan/tyrosine solutions, respectively. Whereas *ϕ*_Trp/Tyr_ is the quantum yield of Trp or Tyr solutions.

### Electrochemical measurements

Cyclic Voltammetry (CV) experiments were performed with 660D electrochemical analyzer (ChenHua, shanghai, China). The glassy carbon electrode (ChenHua, shanghai, China) was used as the working electrode. Ag/AgCl electrode (ChenHua, shanghai, China) was employed as the reference electrode, and a Pt wire (ChenHua, shanghai, China) was used as the auxiliary electrode. Before performing experiment, the working electrode was polished with 0.3 μM alumina. The scan rate was 0.1 V s^−1^. The concentration of Cu^2+^ was 100 μM, and the one of HDSGWEVHH was 200 μM.

### Measurements of electron paramagnetic resonance

The electron paramagnetic resonance (EPR) spectra were recorded by a EMXPLUS spectrometer (Bruker BioSpin, Switzerland). The microwave power was 2.000 mW, the magnetic field modulation frequency was 100.00 kHz and the modulation amplitude was 4.000 G. All the experiments were performed at liquid nitrogen temperatures.

### Nuclear magnetic resonance measurements

HDSGWEVHH solution (5.18 μM) was prepared by directly dissolving the lyophilized solid samples in deuterated water (D_2_O). Then added Cu^2+^ to get HDSGWEVHH (5.18 μM)–Cu^2+^ (2.59 μM) complex. The solutions were transferred into Nuclear Magnetic Resonance (NMR) tubes, and the ^1^H NMR spectra (500 MHz) were recorded by a AVANCE III NMR spectrophotometer (Bruker BioSpin, Switzerland) at room temperature.

### Theoretical calculations for the coordination modes

Firstly, the model of HDSGWEVHH–Cu^2+^ for the computational studies were generated by semiempirical theory calculation (pm7) with the ratio 2 : 1 for the peptide and Cu^2+^. Then, quantum mechanics/molecular mechanics (QM/MM) geometry optimizations for the binding modes were performed with Cu^2+^ and three histidine residues in QM region, while the remainder of the model was treated with MM. All geometer optimizations were carried out on a cluster of 2 pentium Xeon nodes employing the Gaussian 16 suite of programs. The theoretical level b3lyp/6-31G(d) was used for the QM calculation. The universal force field (UFF) and the charges equilibration (QEq) approach were used for the MM region.

### Determination of dissociation constants

The fluorescence intensity changes upon Cu^2+^ binding reflects the amount of peptide–Cu^2+^ complex formed. Thus, dissociation constants, *K*_d_, for peptide–Cu^2+^ were determined from the fluorescence intensity as a function of total free Cu^2+^ concentration, [L], using equation:^38^2
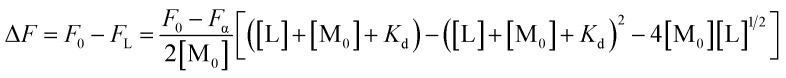
where *F*_0_ and *F*_L_ are the measured fluorescence intensities of the peptide at 356 nm in the absence and presence of the Cu^2+^, respectively. *F*_α_ is the intensity corresponding to the solution in which the peptide is saturated with Cu^2+^ and no further quenching occurs. [M_0_] is the concentration of peptide binding sites (since the actual concentration of binding sites likely differs from the formal peptide concentration, the value of [M_0_] was typically varied in the range 0–2 μM for the fitting procedure). The binding constant, *K*_b_, was taken as the reciprocal of the dissociation constant, *K*_d_.

### The limit of detection

The limit of detection (LOD) was calculated by the data of fluorescence titration experiments. Formula LOD = 3*σ*/*m*, where *σ* is the standard deviation of this blank measurements, which was obtained by the fluorescence intensity of HDSGWEVHH in the absence of Cu^2+^, measured ten times. *m* is the slop of the fluorescence intensity *versus* the concentration of Cu^2+^ ion.^39^ The detection limit of S^2−^ was detected with the same method.

### Determination of free copper concentration released by MT–Cu

HCl and NaOH solutions were used to adjust the pH of the MT–Cu solutions. MT–Cu solutions (100 μM, 1 μL) whose pH were adjusted to different values were used as samples. The concentration of HDSGWEVHH used for determination was 2 μM. The fluorescence intensities of the solutions were recorded.

### Determination of copper concentration in tea sample

The tea samples were dried at 80 °C for 6 h, grounded into powder and sifted through a 80-mesh sieve. The tea powder (0.6 g) was weighted and treated with 12.6 mL HNO_3_ and 1.4 mL HClO_4_ in a digestion tube for 12 h. Then the mixed solution was digested with graphite oven (SH220N, Manon, JiNan, China) followed the digestion procedure as shown in Table S1.† When the temperature reaches 240 °C, 20 mL DI water was added into the tube to remove excess acid solution. After digestion, solutions were cooled to room temperature and diluted into 25 mL with DI water for detecting the Cu^2+^ concentration by peptide probe and atomic absorption spectroscopy (AAS) method. Every sample was repeated three times.

### Determination of copper concentration in water samples

Water samples were filtered with filter (0.45 μm). HDSGWEVHH (10 μL, 200 μM) was added into the HEPES (188 μL, 10 mM, pH 7.4) buffer, and then a filtered real sample (2 μL) was added into the mixture. The fluorescence intensities of the resulting mixtures were recorded. Each experiment was repeated three times.

### Atomic absorption spectroscopy measurements

To assess the accuracy of the method, the Cu^2+^ concentrations in real tea and water samples were also analyzed using an atomic absorption spectrometer (AAS, NOVAA350; Analytik Jena AG, Jena, Germany). The lamp current was set at 3.0 mA, and the spectral bandwidth was operated at 1.4 nm. The analytical wavelength was set at 324.8 nm. The working standard solutions were prepared daily through a stepwise dilution of the standard stock solutions with 0.5% (v/v) nitric acid (HNO_3_). All containers and glassware for the AAS test were cleaned by soaking them in 5 M HNO_3_ for at least 24 h and rinsing three times with DI water prior to use.

### Detection of S^2−^ concentration in real sample

Water samples were filtered with filter (0.45 μm). HDSGWEVHH–Cu^2+^ (40 μL, 10 μM) was added into the HEPES (60 μL, 40 mM, pH 7.4) buffer, and then the filtered sample (100 μL) was added into the mixture. The fluorescence intensities of the resulting mixtures were recorded. Each experiment was repeated three times.

## Results and discussion

### Design and characters of fluorescence peptide probe

Studies on amyloid beta (Aβ) have shown that the three histidine residues provide the coordination sphere to Cu^2+^.^33,40,41^ And tyrosine residue at position 10 of Aβ peptides (Y10) does not contribute to Cu^2+^ binding, but its fluorescence intensity could be decreased when Cu^2+^ bound with the Aβ peptides.^42–45^ According the sequence of Aβ (6–14) (HDSGYEVHH),^46,47^ the modified peptide (HDSGWEVHH) was synthesized for Cu^2+^ determination. The sequence of Aβ(1–16) and our probe were shown in Table 1. Similar as the other reports,^48–51^ histidine (H) residues were selected for binding Cu^2+^ ions and our peptide fluorescence probe (HDSGWEVHH) is organic fluorophore label free and easily synthesized with amino acid residues.

**Table 1:** Sequence of Aβ(1–16) and fluorescent probe

Peptide	Sequence
Aβ(1–16)	DAEFRHDSGYEVHHQK
Probe	HDSGWEVHH

**Fig. 2:**
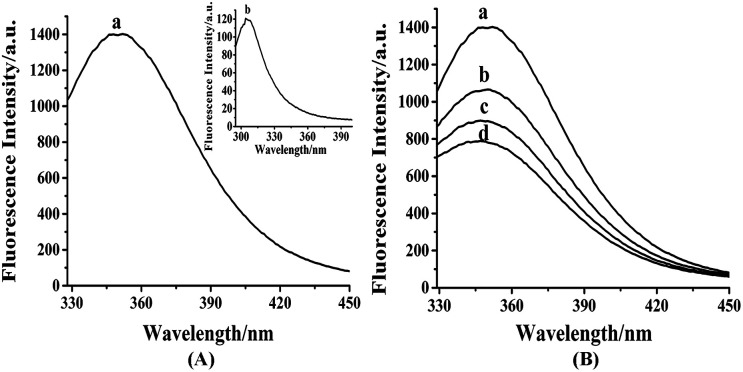
(A) Fluorescence spectra of HDSGWEVHH and Aβ(1–16) with the same excitation wavelength at 280 nm. (B) Fluorescence spectra of HDSGWEVHH in the absence (curve a) and presence of 0.49 (curve b), 0.74 (curve c) and 1 μM (curve d) Cu^2+^. The spectra were obtained in 10 mM HEPES buffer (pH 7.4), and the concentrations of Aβ(1–16) and HDSGWEVHH were both at 2 μM.

As shown in Fig. S3,† the maximum absorption peak of HDSGWEVHH was around 280 nm. Thus 280 nm was chosen in our work as the excitation wavelength (*λ*_ex_) for fluorescence emission determination. As shown in Fig. 2A, the emission wavelength of Aβ(1–16) is at 304 nm (Fig. 2A, line b), while the one of HDSGWEVHH is at 356 nm (Fig. 2A, line a). And the fluorescence intensity of HDSGWEVHH is around 1380, which is around 11 times higher than the one of Aβ(1–16). This is due to the quantum yield (*ϕ*) value of HDSGWEVHH was 0.21 (*cf. ϕ* value calculated with eqn (1)), which is much stronger than the one of Aβ(1–16) (0.04) (*cf. ϕ* value calculated with eqn (1)).

With the addition of Cu^2+^, the fluorescence intensity of probe decreases inversely (Fig. 2B). This behavior was highly comparable to the other Cu^2+^-bound peptides, such as amyloid beta (1–42).^52^ This is because the nitrogen of the imidazole ring of histidine provides the coordination sphere to Cu^2+^.^33^ Tyrosine (in Aβ(1–16)) or tryptophane (in our probe) is not involved in the coordination, but their fluorescence could be quenched by the interaction between peptide and Cu^2+^. As shown in Fig. 2B, the fluorescence intensity of probe is highly dependent on copper concentration. These results indicated that HDSGWEVHH can be considered as a fluorescent probe for determining the concentration of Cu^2+^.

**Fig. 3:**
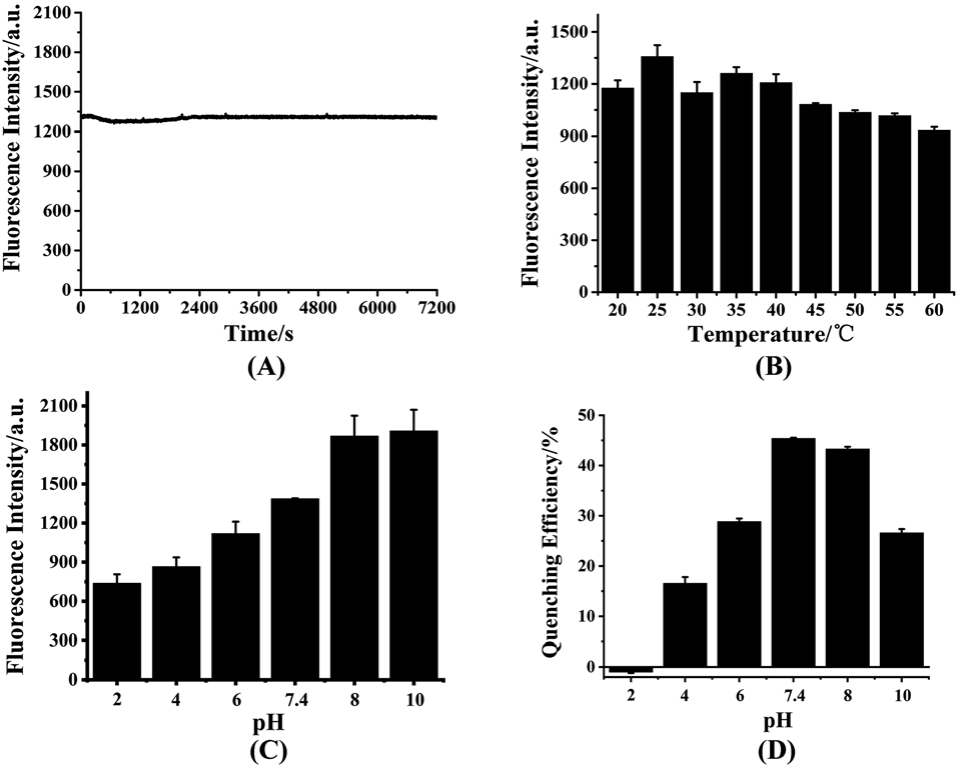
Fluorescence intensity of HDSGWEVHH at different conditions, including different scanning time (A), different temperature (B), different pH (C) and the quenching efficiency of Cu^2+^ to HDSGWEVHH (D). The concentration of HDSGWEVHH was 2 μM, and the one for Cu^2+^ was 1 μM.

To investigate the interference of the environment to the fluorescence intensity of HDSGWEVHH, the fluorescence intensity were detected at different conditions. As shown in Fig. 3A, with 2 h scanning, the fluorescence intensity of the probe kept stably, which indicated the probe has a good stability. This result also suggested that the peptide had not aggregated during the scanning time, which is consistent with the Aβ(1–16) peptide.^53^ The fluorescence intensity of probe which incubated at different temperature was also recorded (Fig. 3B). At 25 °C, the highest fluorescence intensity was obtained. At the higher temperature (above 25 °C), the lower fluorescence intensity was obtained. This is probably due to that with the increase of temperature, the probability of molecular collision was increased. And the non-radiative transition occurred, which resulted in the decreased fluorescence intensity.^54^ As shown in Fig. 3C, when the pH value was in the range of 2 to 8, fluorescence intensity of the probe increased with the pH raise. When the pH value was above 8, the fluorescence intensity reached the highest value and kept stable. The influence of pH on the fluorescence intensity is probably due to the alteration of the molecular orbital of the excitable electrons.^55^ This also could be the consequence of ionization status of the fluorescent molecules at different pH.^55^ As shown in Fig. 3D, when the pH value was around 7.4 or 8.0, the fluorescent quenching efficiency was reached the highest value (around 45%). One possible explanation was that at pH 7.4 or 8.0, the ionization status of HDSGWEVHH could provide a more stable and tight coordination sphere binding to Cu^2+^. Owing to the 7.4 is more closer to the neutral pH and the normal human fluid pH, pH 7.4 was selected for the assays.

### Complexation mechanism of HDSGWEVHH–Cu^2+^ complex

**Fig. 4:**
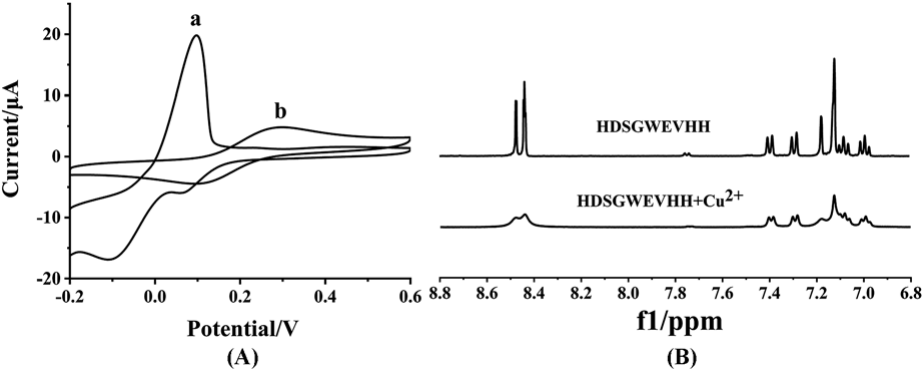
(A) CV spectra of 100 μM Cu^2+^ solution (a) and 200 μM HDSGWEVHH/100 μM Cu^2+^ mixture (b). (B) ^1^H NMR spectra of HDSGWEVHH (5.18 μM) in D_2_O in the absence of Cu^2+^ and presence of Cu^2+^ (2.59 μM).

To investigate the mechanism of the copper complexation and oxidation state of Cu, ^1^H NMR, EPR, CV and computational studies were employed.^56^ As shown in Fig. 4A, Cu^2+^ in solution shows peaks at 0.098 mV, 0.066 mV and −0.110 mV *vs.* Ag/AgCl (Fig. 4A-a) It indicated that in the presence of oxygen, the Cu^+^ reduced from free Cu^2+^ and consequent Cu^+^ is further reduced to Cu^0^.^57,58^ But the mixture of HDSGWEVHH/Cu^2+^ shows peaks at 0.300 mV and 0.093 mV *vs.* Ag/AgCl. CV results indicated the formation of a electrochemically stable complex in HDSGWEVHH/Cu^2+^ solution, but do not provide any conclusive structural information. Analysis of EPR spectra of frozen solutions obtained that copper exists in +2 oxidation state both in the absence and presence of HDSGWEVHH (Fig. S4†). ^1^H NMR analysis of HDSGWEVHH solution shows signals that arise at 8.60–6.90 ppm, which were attributed to the protons of the aromatic region. Thus these signals were assigned to the protons of histidine imidazole rings and tryptophone benzpyrole rings.^59^ With the addition of Cu^2+^, the peaks at 8.60–6.90 ppm were broadening (Fig. 4B). These results are consistant with other studies that metal binding with the peptide could cause a number of ^1^H NMR resonances, resulting in broadening signals.^60,61^ Our results indicated that the protons of histidine imidazole rings and tryptophone benzpyrole rings were influenced by Cu^2+^ addition. But the coordination mode is also not clear.

**Fig. 5:**
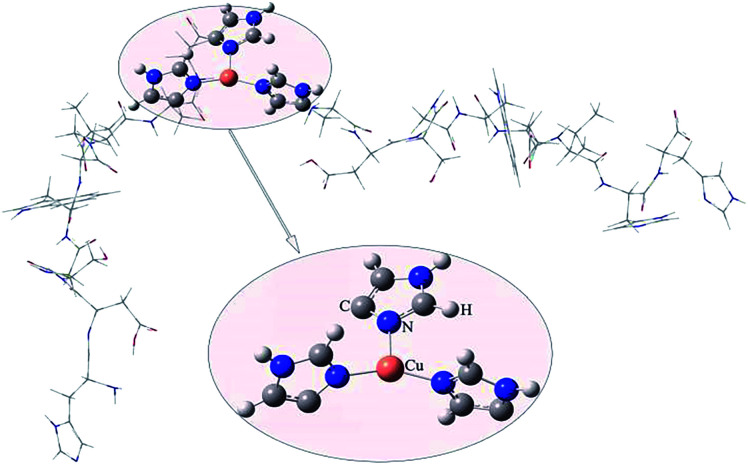
QM/MM-optimized molecular geometries of HDSGWEVHH–Cu^2+^. The balls denote the Cu^2+^ and the ligands included in the QM region. The thin gray wireframe donates the MM region.

To investigate the coordination mode of HDSGWEVHH–Cu^2+^ complex, computational studies were employed for theoretical calculation. As shown in Fig. 5, the molar radio of HDSGWEVHH to Cu^2+^ in the complex was 2 : 1, and a T-shaped three-coordinate mode was found by the QM/MM calculation for HDSGWEVHH–Cu^2+^ complex. In this T-shaped mode, Cu^2+^ is coordinated by three histidines, of which two are from C-terminate of one HDSGWEVHH chain, and another is from N-terminate of the other HDSGWEVHH chain. This result is consistent with recent researches.^62–65^

### Dissociation constant detection of HDSGWEVHH–Cu^2+^ complex

**Fig. 6:**
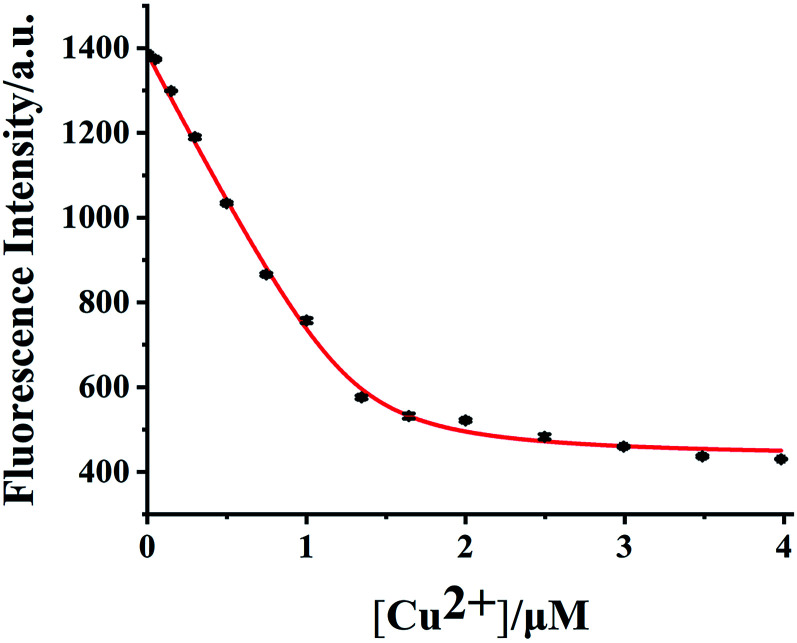
Determination of *K*_d_ using the quenching of the probe fluorescence by Cu^2+^ at pH 7.4. The squares are experimental data, and the curve is a nonlinear-least-squares regression fit with eqn (2) with a probe concentration of 2.0 μM (*R*^2^ = 0.998).

To calculate the dissociation constant (*K*_d_) of HDSGWEVHH–Cu^2+^, the fluorescence intensity at 356 nm for HDSGWEVHH in the presence of different concentrations of Cu^2+^ was obtained. When Cu^2+^ concentration is greater than 1.0 μM, the HDSGWEVHH fluorescence intensity turned out to be stable (as shown in Fig. 6). It is suggested that the molar ratio of the HDSGWEVHH to Cu^2+^ in the complex is 2 : 1 which is consistent with the results of computational calculation results (Fig. 5). Meanwhile, the dissociation (*K*_d_) constant of HDSGWEVHH peptide to Cu^2+^ was analyzed by a nonlinear-least-squares regression with eqn (2),^38^ depending on the fluorescence intensity changes of HDSGWEVHH as a function of Cu^2+^ concentration (Fig. 6). The peptide concentration used for the binding constant measurement was 2 μM, and free Cu^2+^ concentration was fixed within 0–1 μM. The calculated dissociation (*K*_d_) and binding (*K*_b_) constants of HDSGWEVHH peptide to Cu^2+^ at pH 7.4 were 5.8 × 10^−8^ M and 1.72 ×10^8^ M^−1^, respectively. The calculated *K*_b_ is two order higher than the one of Aβ(1–16) (1 × 10^6^ M^−1^).^38^ This is probably due to that comparing with Aβ(1–16),^66^ the lack of amino acid residues at the N and C terminals, which resulted in the exposing of HDSGWEVHH (His) coordination site directly to copper (Fig. 5). Three histidine residues which from two different HDSGWEVHH molecules provided more tight coordination sphere to Cu^2+^ than Aβ(1–16).

**Fig. 7:**
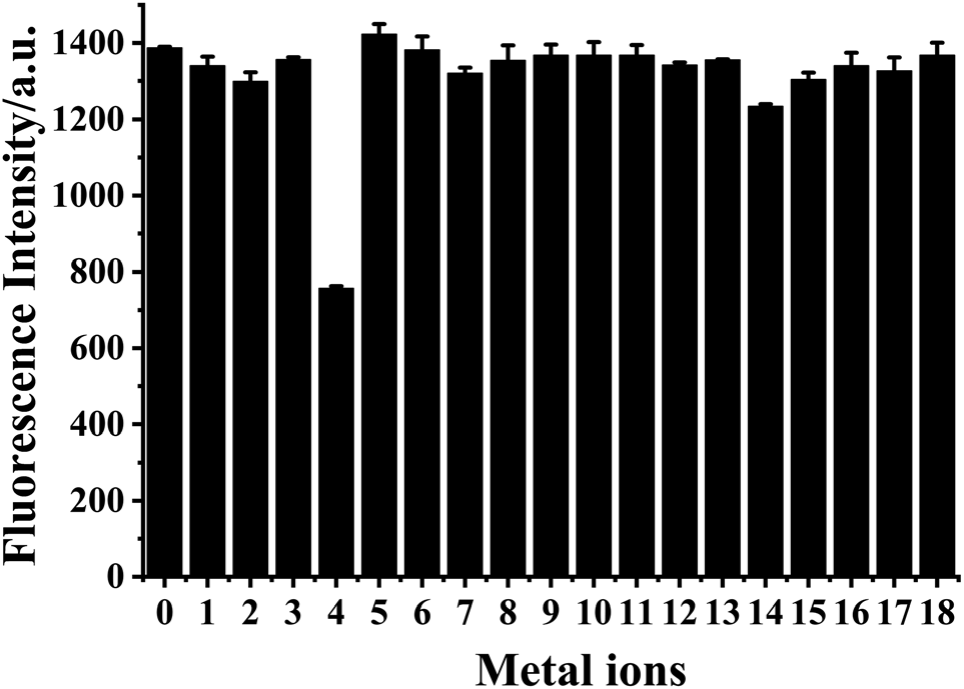
Selectivity of HDSGWEVHH probe to Cu^2+^ with respect to common metal ions. Fluorescence intensity of HDSGWEVHH solution in the absence of metal ions (0) and in the presence of metal ions: Cr^3+^ (1), Fe^3+^ (2), Al^3+^ (3), Cu^2+^ (4), Fe^2+^ (5), Zn^2+^ (6), Pb^2+^ (7), Co^2+^ (8), Ca^2+^ (9), Mn^2+^ (10), Mg^2+^ (11), Ba^2+^ (12), Cd^2+^ (13), Hg^2+^ (14), Ni^2+^ (15), Cs^+^ (16), Na^+^ (17), and K^+^ (18). The concentrations of HDSGWEVHH, Al^3+^, Ba^2+^, Cd^2+^, Hg^2+^, Ni^2+^ and Pb^2+^ were 2 μM, the one of Cu^2+^ was 1 μM, and the concentrations of other metal ions were 100 μM. The relative standard deviations, shown as error bars, vary from 0.22 to 2.94%.

The selectivity of HDSGWEVHH to Cu^2+^ was assessed against common metal ions, such as Cr^3+^, Fe^3+^, Al^3+^, Cu^2+^, Fe^2+^, Zn^2+^, Pb^2+^, Co^2+^, Ca^2+^, Mn^2+^, Mg^2+^, Ba^2+^, Cd^2+^, Hg^2+^, Ni^2+^, Cs^+^, Na^+^, and K^+^ (Fig. 7). Among these ions, only Cu^2+^ displayed noticeable fluorescence quenching. Our results therefore demonstrate that HDSGWEVHH is a viable probe for determining Cu^2+^ concentrations in water samples.

### Selectivity and fluorescence responses of HDSGWEVHH to Cu^2+^

**Fig. 8:**
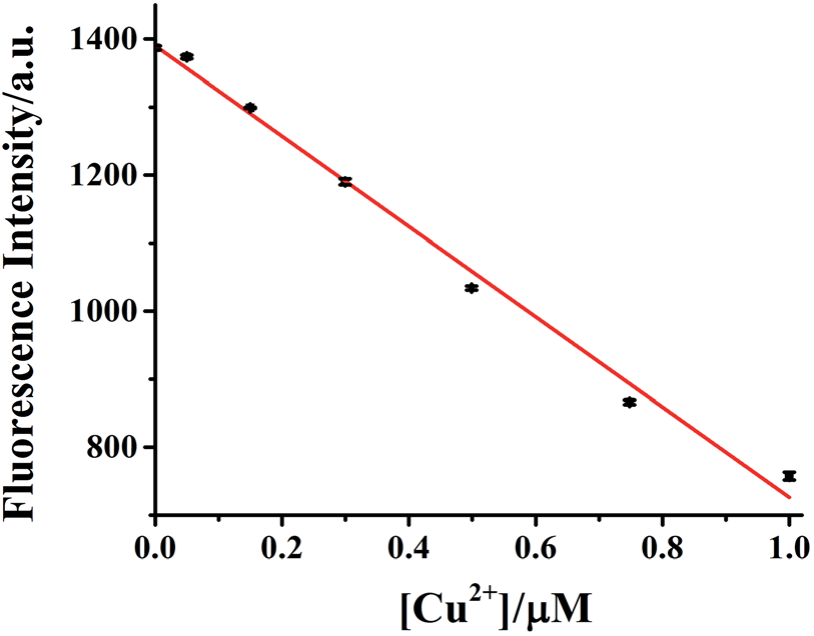
Calibration curve of 2 μM HDSGWEVHH for Cu^2+^ concentration determination. The linear regression yields *y* = −663.91[Cu^2+^] + 1390 (in μM). A HEPES (10 mM, pH 7.4) buffer was used. The error bars were computed from at least three replicates.

The dependence of HDSGWEVHH fluorescence intensity on Cu^2+^ concentration was also investigated. As shown in Fig. 8, when the concentration of HDSGWEVHH was 2 μM, between 50 nM and 1.0 μM, a linear dependence (*R*^2^ = 0.9928) was observed. Increase the concentration of the probe from 2 μM to 8 μM, the similar trend was obtained (Fig. S5†). And the linear range of 8 μM probe was from 150 nM to 4.0 μM. It indicated that the linear range of the probe is concentration dependent. The detection limit (3*σ*/*m*) of HDSGWEVHH for Cu^2+^ detection was estimated to be 16 nM (*n* = 10) when the probe concentration was 2 μM. The lower detection limit is due to the high binding affinity (1.72 × 10 ^8^ M^−1^) of HDSGWEVHH to Cu^2+^ (Fig. 6).

### Copper determination in real samples

To gauge the ability of HDSGWEVHH probe for real sample analyses, free Cu^2+^ concentrations released from MT were detected. And Cu^2+^ concentrations in different water and tea samples were also measured with HDSGWEVHH.

MTs are vital for essential metal (*e.g.* Cu) regulation and heavy metal detoxification in human body. MTs bind tightly with Cu *via* the thiolate groups of their abundant cysteine residues (∼30% of the total amino acid residues), thereby forming thiolate–Cu^+^ clusters at natural pH.^67^

Free Cu^2+^ concentrations released from the MT–Cu complex at different pH were measured with HDSGWEVHH. As shown in Table 2, at neutral pH, Cu is bound to MT tightly, and no free Cu^2+^ was detected by HDSGWEVHH. When the pH was adjusted to 0.5, Cu^+^ was released from the MT–Cu complex and quickly oxidized by O_2_ in solution to Cu^2+^. As shown in Table 2 (pH 0.5), free Cu^2+^ concentration was determined to be 1.00 μM by HDSGWEVHH probe. It indicated that at low pH, Cu^+^ bound by MT is completely released, which is consistent with other reports.^21,68^

**Table 2:** Measurement of released Cu^2+^ from 1 μM MT–Cu complex at different pH

pH	7.4	6.0	0.5
[Cu^2+^]/μM	0.00	0.00	1.00 ± 0.01

The concentrations of Cu^2+^ in tap water, water in Xiangjiang river and tea were determined as 0.137 mg kg^−1^, 0.515 mg kg^−1^, and 15.730 mg kg^−1^ (Table 3), which were consistent with the concentrations evaluated with AAS, suggesting that the method used in this study was accurate and could be straightforwardly implemented for the assay of free Cu^2+^ in water and food samples.

**Table 3:** Measurement of Cu^2+^ in different real samples with AAS and fluorescent probe

Sample	Cu^2+^ determined (mg kg^−1^)	
	AAS	HDSGWEVHH
Tap water	0.136 ± 0.001	0.137 ± 0.002
Xiangjiang river water	0.512 ± 0.002	0.515 ± 0.001
Tea	15.600 ± 0.007	15.730 ± 0.008

### Sulfide determination by HDSGWEVHH–Cu^2+^ complex

**Fig. 9:**
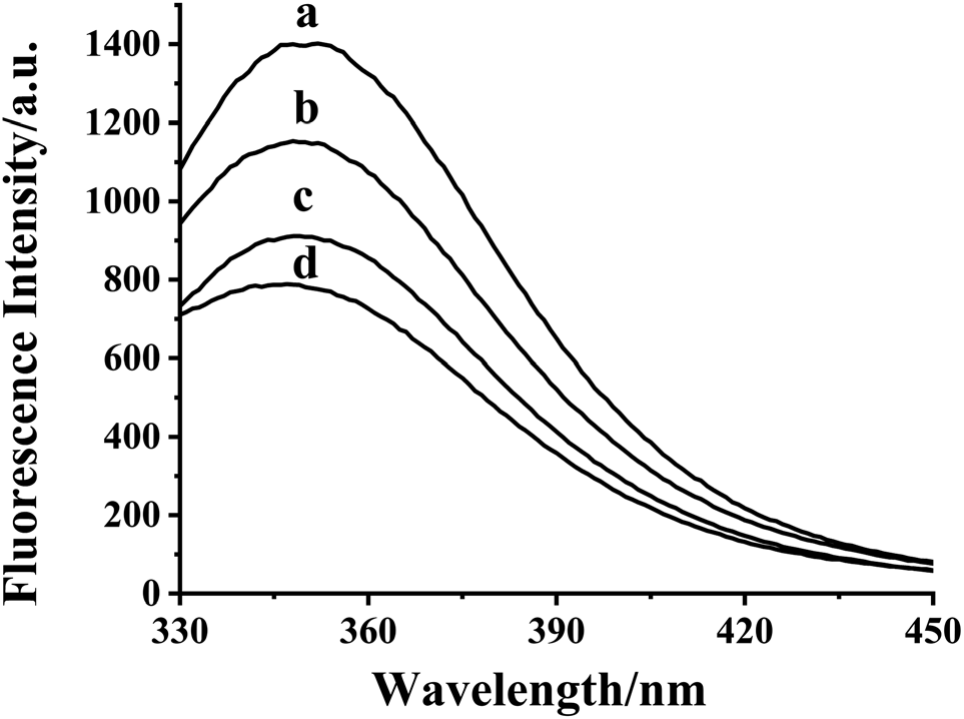
Fluorescence spectra of 2 μM HDSGWEVHH (a) and 2 μM HDSGWEVHH/1 μM Cu^2+^ mixture in the absence (d) and presence of 0.30 (c) and 0.85 μM (b) S^2−^. All the spectra was obtained in 10 mM HEPES buffer (pH 7.4).

As shown in Fig. 9, the fluorescence intensity of HDSGWEVHH–Cu^2+^ mixture increases with the S^2−^ concentration. Owing to the much higher binding affinity of Cu^2+^ to S^2−^ (*K*_a_ = 1.6 × 10^37^ M^−1^)^55^ than to HDSGWEVHH (1.72 × 10^8^ M^−1^), S^2−^ can deprive Cu^2+^ from the HDSGWEVHH–Cu^2+^ complex, and release the HDSGWEVHH into solution, which results in the fluorescence recovery. The results indicated that the HDSGWEVHH–Cu^2+^ complex could be considered as the determination probe for S^2−^. Owing to the fast precipitation reaction rate, the fluorescence intensity in HDSGWEVHH–Cu^2+^ solution was recovered immediately with the addition of S^2−^. While, some organic fluorescent probes (*e.g.* benzopyran derivative compounds) for S^2−^ detection need a long reaction time (30–60 minutes) to get the changes of fluorescence.^26,47,69^

### Selectivity and fluorescence response of HDSGWEVHH–Cu^2+^ complex to sulfide

**Fig. 10:**
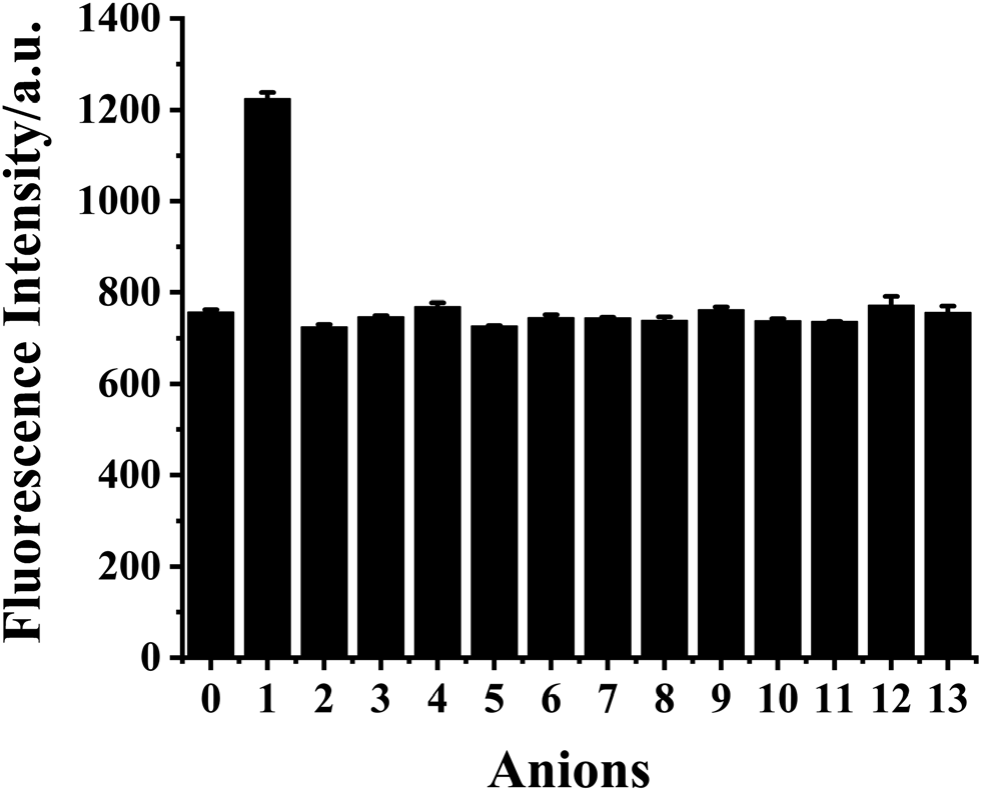
Fluorescence intensity of HDSGWEVHH (2 μM)–Cu^2+^ (1 μM) complex solution in the absence of anions (0) and in the presence of S^2−^ (1), S_2_O_3_^2−^ (2), SO_4_^2−^ (3), SO_3_^2−^ (4), HSO_3_^−^ (5), PO_4_^3−^ (6), HCO_3_^−^ (7), NO^2−^ (8), OAc^−^ (9), F^−^ (10), Cl^−^ (11), Br^−^ (12), and I^−^ (13). The concentration of anions was 2 μM. The relative standard deviations, shown as error bars, vary from 0.76 to 13.28%.

The selectivity of HDSGWEVHH–Cu^2+^ complex to S^2−^ with respect to common anions (Fig. 10) was investigated. The HDSGWEVHH–Cu^2+^ complex displayed a remarkable selectivity to S^2−^. When S^2−^ was added to the HDSGWEVHH–Cu^2+^ solution, the HDSGWEVHH fluorescence was quantitatively recovered. The fluorescence intensity of the HDSGWEVHH–Cu^2+^/S^2−^solution was around 1220, which is around 1.6 times than that of HDSGWEVHH–Cu^2+^ solution. However, the fluorescence intensity of other anions solutions was similar with that of HDSGWEVHH–Cu^2+^ solution. Notice that the selectivity was not affected by other sulfur-containing anions such as S_2_O_3_^2−^, SO_4_^2−^, SO_3_^2−^, and HSO^3−^.

**Fig. 11:**
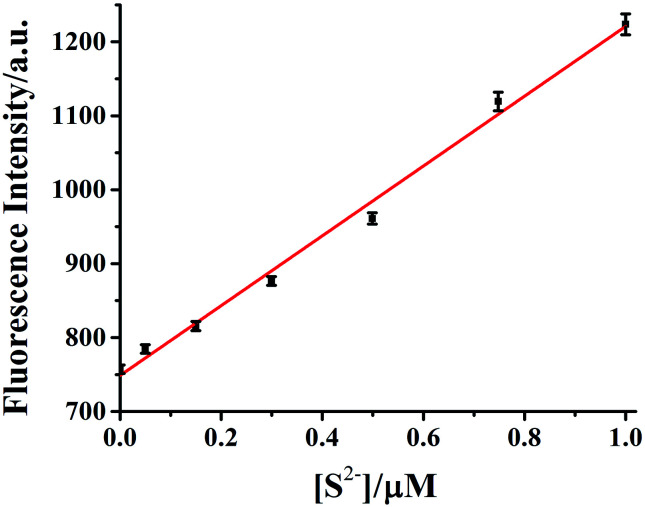
Calibration curve of HDSGWEVHH (2 μM)–Cu^2+^ (1 μM) for S^2−^ concentration determination. A HEPES (10 mM, pH 7.4) buffer was used and the linear regression yields signal = 748.06 + 472.17 [H_2_S] (in μM). The error bars were computed from at least three replicates.

The dependence of fluorescent changes of HDSGWEVHH–Cu^2+^ on S^2−^ concentrations was monitored. As shown in Fig. 11, a linear calibration curve was obtained within 50 nM to 1.0 μM of S^2−^ (*R*^2^ = 0.9933). When the S^2−^ concentration is higher than that of Cu^2+^, the plot was found to reach a plateau (data not shown). The plateau suggests that all of the Cu^2+^ bound by HDSGWEVHH can be extracted by S^2−^ to precipitate as CuS. But there was also little peptide was precipitated with the ions, which resulted in the little decrease of the fluorescence intensity of HDSGWEVHH. The detection limit (3*σ*/*m*) of the method for S^2−^ in buffer was estimated to be 19 nM, which was much lower than the maximum allowable level of S^2−^ (15 μM) in drinking water set by the WHO.^15^

### Sulfide determination in real water samples

To gauge the ability of HDSGWEVHH–Cu^2+^ probe for real sample analyses, concentration of S^2−^ in water sample was measured with HDSGWEVHH. The concentrations of S^2−^ in water was determined as 3.3 μM, which was consistent with other reports,^70,71^ suggesting that the method used in this study was accurate and could be straightforwardly implemented for the S^2−^ concentration determination in water samples.

## Conclusions

A mutant amyloid peptide, HDSGWEVHH, has been demonstrated as a highly sensitive and selective fluorescent probe to free Cu^2+^ and S^2−^ present in food and aqueous samples. The concentration of Cu^2+^ can be detected by monitoring the decreases in the fluorescence intensity of HDSGWEVHH, which were owing to the high affinity (*K*_b_ = 1.72 × 10 ^8^ M^−1^) of the mutant peptide *via* binding of Cu^2+^ through the histidine residues. The method is highly sensitive and selective with a detection limit of 16 nM for Cu^2+^. In addition, the generated HDSGWEVHH–Cu^2+^ complex ensemble can serve as a S^2−^ sensor because of the quenched fluorescence could be recovered with S^2−^ addition. The recovery of fluorescence intensity was due to the CuS formation, which resulted in HDSGWEVHH released from HDSGWEVHH–Cu^2+^ complex. With the fluorescence recovery, the S^2−^ concentrations measured with a detection limit of 19 nM. Meanwhile, concentrations of Cu^2+^ released from MT–Cu complex, concentrations of Cu^2+^ in food samples and S^2−^ in the real water sample were detected with the HDSGWEVHH probe. Our results demonstrated that peptide based fluorescent probe can serve as a label free, easily synthesized, non-cytotoxicity fluorescence probe for facile and sensitive analyses of Cu^2+^ and S^2−^ in food and aqueous samples.

## Conflicts of interest

There are no conflicts to declare.

## Supplementary Material

RA-011-D0RA08788B-s001
