# Supplementary material for: A label-free fluorescent peptide probe for sensitive and selective determination of copper and sulfide ions in aqueous systems
Source: RSC Adv. 2021 Feb 17;11(13):7426–35. doi: 10.1039/d0ra08788b (PMC8694936; doi:10.1039/d0ra08788b)
Supplement: RA-011-D0RA08788B-s001 [file RA-011-D0RA08788B-s001.pdf]

Electronic Supplementary Material (ESI) for RSC Advances.
This journal is © The Royal Society of Chemistry 2021

Supplementary Information

A Label-Free Fluorescent Peptide Probe for Sensitive and Selective Determination of Copper and Sulfide ions in Aqueous System

Yadan Zhang,[a] Yunhui Cai,[a] Yonghui He,[b] Qinlu Lin,[a] Jiali Ren,[a] Dongsheng Cao[c] and Lin Zhang *[a]

[a.] *National Engineering Laboratory for Rice and Byproduct Deep Processing, Hunan Key Laboratory of Processed Food For Special Medical Purpose, Hunan Key Laboratory of Forestry Edible Resources Safety and Processing, School of Food Science and Engineering, Center South University of Forestry and Technology, Changsha 410004, P.R. China. E-mail: zhanglin840514@126.com*

[b.] *Key Laboratory of Chemistry in Ethnic Medicinal Resources, State Ethnic Affairs Commission & Mini stry of Education, Yunnan Minzu University, Kunming, Yunnan, 650500, P.R. China*

[c.] *Xiangya School of Pharmaceutical Science, Central South University, Changsha 410083, P.R. China*

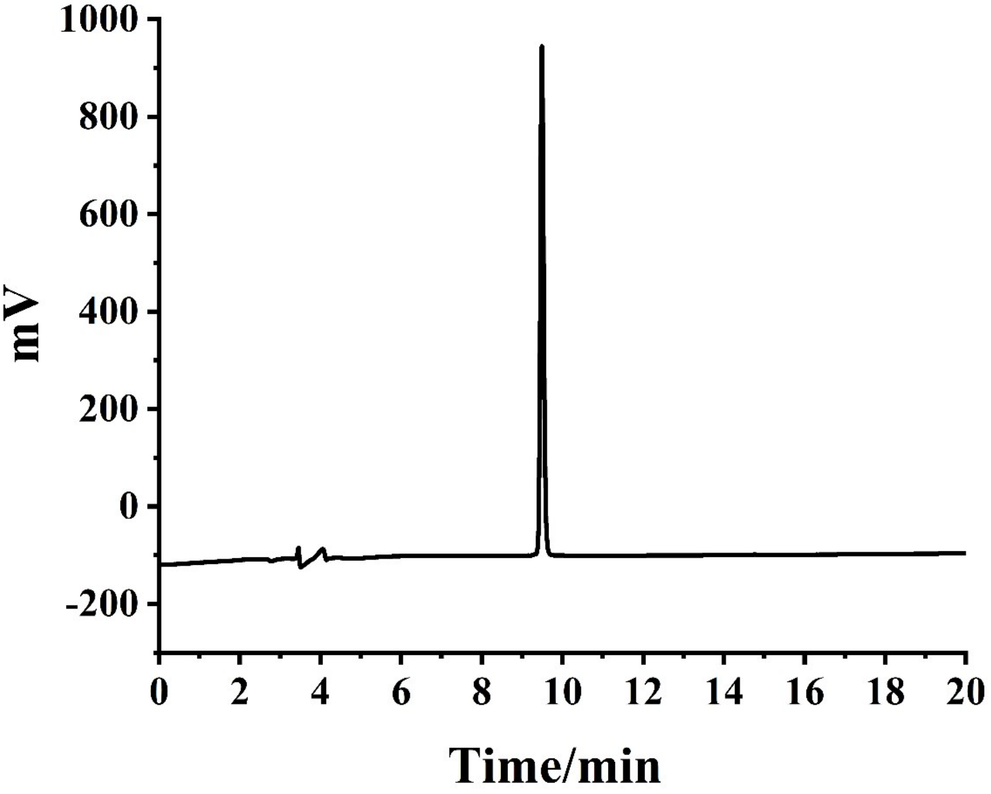

Fig. S1 HPLC spectrum of HDSGWEVHH.

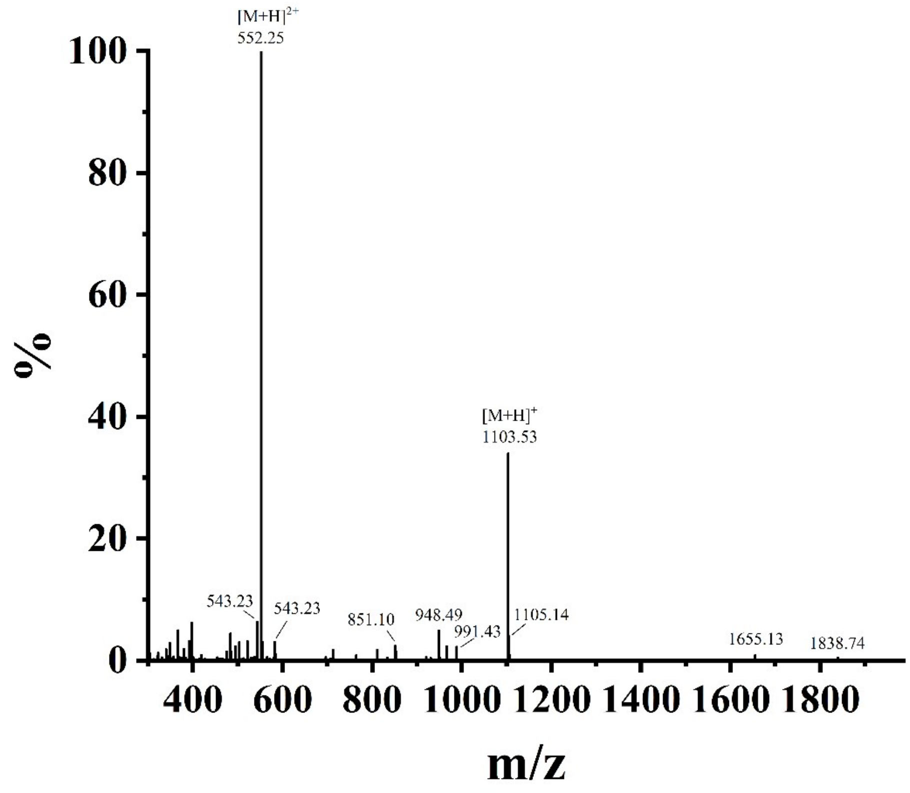

Fig. S2 LC-MS spectrum of the HDSGWEVHH.

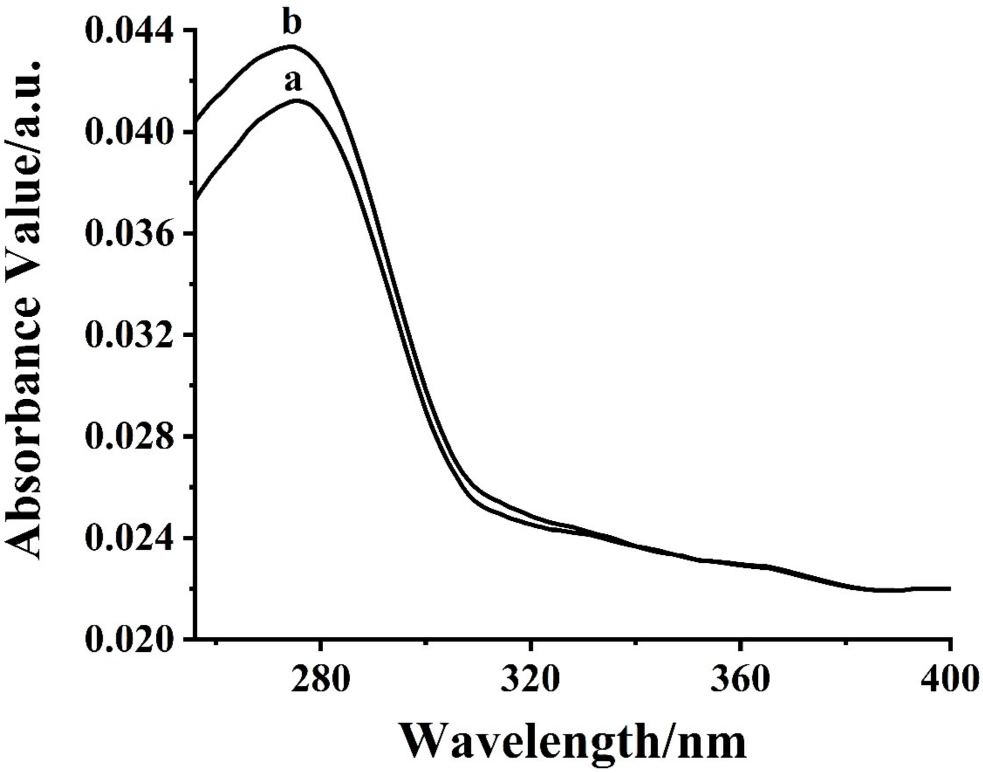

Fig. S3 UV-Vis absorption spectra of HDSGWEVHH in absence of Cu^{2+} (line a) and presence of Cu^{2+} (line b). The spectra were obtained in 10 mM HEPES buffer (pH 7.4). The concentration of HDSGWEVHH was at 2 µM, and the one of Cu^{2+} was 1µM.

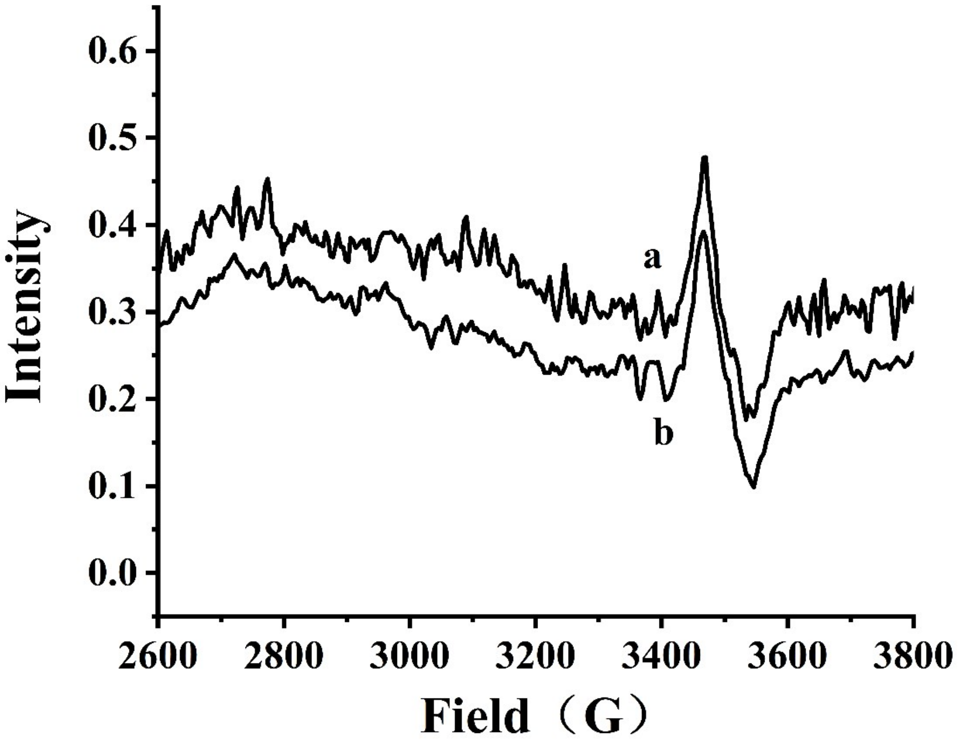

Fig. S4 EPR spectra of the 2μM HDSGWEVHH solution (a) and 2 μM HDSGWEVHH/1 μM Cu^{2+} mixture (b).

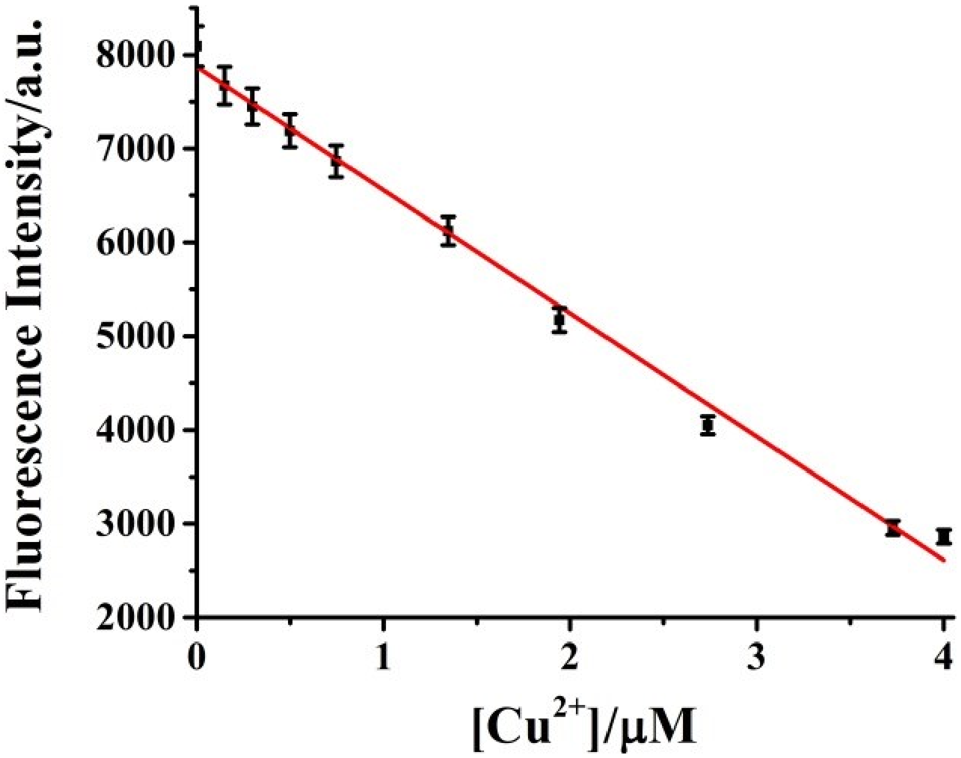

Fig. S5 Calibration curve of 8 μM HDSGWEVHH for Cu^{2+} concentration determination. The linear regression yields y = -1315.3[Cu] + 7874.1 （R^2 = 0.9947） (in μM). A HEPES (10 mM, pH 7.4) buffer was used. The error bars were computed from at least three replicates.

Table S1 Stepped temperature program of graphite digestion procedure

Temperature/℃	Time/min
95	30
120	60
160	60
190	60
240	60
